# Transcriptome analysis of *Loxosceles laeta *(Araneae, Sicariidae) spider venomous gland using expressed sequence tags

**DOI:** 10.1186/1471-2164-9-279

**Published:** 2008-06-12

**Authors:** Matheus de F Fernandes-Pedrosa, Inácio de LM Junqueira-de-Azevedo, Rute M Gonçalves-de-Andrade, Leonardo S Kobashi, Diego D Almeida, Paulo L Ho, Denise V Tambourgi

**Affiliations:** 1 Laboratório de Imunoquímica, Instituto Butantan, Av. Prof. Vital Brazil, 1500, CEP 05503-900, São Paulo, SP, Brazil; 2 Centro de Biotecnologia, Instituto Butantan, Av. Prof. Vital Brazil, 1500, CEP 05503-900, São Paulo, SP, Brazil; 3 Departamento de Farmácia, Universidade Federal do Rio Grande do Norte, Av. Gal. Cordeiro de Farias, s/n, CEP 59010-180, Natal, RN, Brazil

## Abstract

**Background:**

The bite of spiders belonging to the genus *Loxosceles *can induce a variety of clinical symptoms, including dermonecrosis, thrombosis, vascular leakage, haemolysis, and persistent inflammation. In order to examine the transcripts expressed in venom gland of *Loxosceles laeta *spider and to unveil the potential of its products on cellular structure and functional aspects, we generated 3,008 expressed sequence tags (ESTs) from a cDNA library.

**Results:**

All ESTs were clustered into 1,357 clusters, of which 16.4% of the total ESTs belong to recognized toxin-coding sequences, being the Sphingomyelinases D the most abundant transcript; 14.5% include "possible toxins", whose transcripts correspond to metalloproteinases, serinoproteinases, hyaluronidases, lipases, C-lectins, cystein peptidases and inhibitors. Thirty three percent of the ESTs are similar to cellular transcripts, being the major part represented by molecules involved in gene and protein expression, reflecting the specialization of this tissue for protein synthesis. In addition, a considerable number of sequences, 25%, has no significant similarity to any known sequence.

**Conclusion:**

This study provides a first global view of the gene expression scenario of the venom gland of *L. laeta *described so far, indicating the molecular bases of its venom composition.

## Background

Envenomation by spiders of the *Loxosceles *species (brown spiders) can produce severe clinical symptoms, including dermonecrosis, thrombosis, vascular leakage, hemolysis and persistent inflammation [1].

*Loxosceles *is the most poisonous spider in Brazil and children, who develop the most severe systemic effects after envenomation, nearly always die. At least three different *Loxosceles *species of medical importance are known in Brazil – *L. intermedia, L. gaucho, L. laeta *– and more than 3,000 cases of envenomation by *L. intermedia *alone are reported each year. In North America, several *Loxosceles *species, including *L. reclusa *(brown recluse), *L. apachea, L. arizonica, L. unicolor, L. deserta and L. bonetti *are known to be the principal cause of numerous incidents of envenomation [2-5]. In South Africa, *L. parrami *and *L. spinulosa *are responsible for cutaneous loxoscelism [6] and, in Australia, a cosmopolitan species, *L. rufescens*, is capable of causing ulceration in humans.

In the site of the envenomation, there is initially only a minor discomfort. It begins as an expanding area of oerythema and oedema. A centrally located necrotic ulcer often forms 8–24 h after envenomation [7,8]. Extensive tissue destruction occurs and the ulcer takes many months to heal; in extreme cases, debridement or skin grafting can be necessary. The lesions are remarkable considering that *Loxosceles *spiders inject only a few tenths of a microliter of venom containing no more than 30 μg of protein.

Mild systemic effects induced by envenomation, such as fever, malaise, pruritus and exanthema are common, whereas intravascular hemolysis and coagulation, sometimes accompanied by thrombocytopenia and renal failure, occur in approximately 16% of the victims [1-4,9-11]. Although systemic loxoscelism is less common than the cutaneous form, it is the main cause of death associated with *Loxosceles *envenomation. Most of the deaths occur in children and are related to the South American species *L. laeta *[1]. Due to our limited understanding of the venom's mechanism of action, effective treatment is currently not available.

We have purified and cloned several sphingomyelinases D (SMase D) from *L. laeta *and *L. intermedia *venoms and shown that they are responsible for all the main local and systemic effects induced by whole venom [12-14]. SMase D cleaves sphingomyelin into choline and ceramide 1-phosphate and has intrinsic lysophospholipase D activity toward LPC [15]. The venoms of various *Loxosceles *species contain several functionally active isoforms of the SMase D, the identity varying from 40–90% [5,13,14].

Even though the venom of *Loxosceles *sp spiders is being well studied, there is little information about the spider venom gland at the molecular level and a limited number of annotated *Loxosceles *spider nucleotide sequences, currently deposited in the public databases. Analysis of expressed sequence tags (ESTs) has been utilized as an efficient approach for gene discovery, expression profiling [16,17] and development of resources useful for functional genomics studies. Thus, the aim of our study was to investigate the molecular complexity of the *Loxosceles *venomous gland, by analyzing the repertoire of transcripts using, as strategy, expressed sequence tags.

## Results and Discussion

### Overview of EST from the venom gland of L. laeta

After discarding the poor-quality sequences, 3,008 high-quality ESTs were used to analyze gene expression profile in the venom gland of *L. laeta*. ESTs were clustered into 1,357 clusters, of which 326 correspond to 'contigs' and 1031 to 'singlets'. Therefore, these clusters were considered as putative unigenes, although some of them could still represent different segments of the same gene. All sequences data reported in this paper have been submitted into the public database [GenBank: EY188373 – EY189729].

Sequence clusters were named as LLAE0001c to LLAE0326c, for clusters with more than one read assembled, or as LLAE0327s to LLAE1357s, for clusters containing only one sequence read. When compared to data present on GenBank and dbEST, it was observed that from the 1,357 clusters (3,008 clones), 751 exhibited significant similarities to known cDNA and protein sequences. This corresponds to 1930 clones (64.2%); the remaining 741 clones (13.5%) were not identified. Sixty four clusters (337 clones), exclusively matching with mitochondrial DNAs, mRNAs and ribosomal RNAs, were also found and excluded from the quantitative analyses.

**Figure 1:**
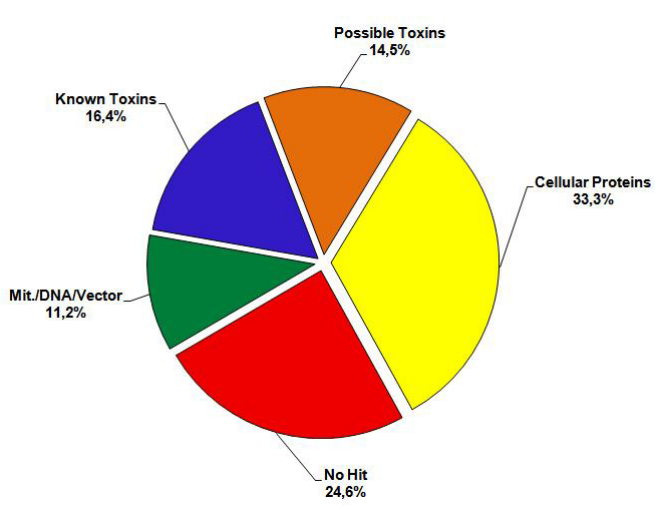
**Functional classification of the transcripts from *Loxosceles laeta *venom glands.** Graph showing the relative proportion of different types of transcripts: 'Known Toxins', 'Possible Toxins' and 'Cellular Proteins', and no-match sequences, 'No Hit'.

The identified clusters were organized in three categories, *e.g*., "known toxins" for clusters coding for proteins that are similar to well-known toxins from spider venoms; "possible toxins" for clusters coding for molecules with a probable toxic activity but with sequences not yet observed in spider venoms; and "cellular proteins" for those coding proteins related to cellular functions, without evidence of being toxins. Figure 1 shows that 'known toxins' correspond to 16.4% of all cDNAs (93 clusters with 494 clones) and 25.6% of the identified messages, while 'possible toxins' to 14.5% of all cDNAs (117 clusters with 435 clones) and 22.5% of the identified messages. The 'cellular proteins' represent 33.3% of the total number of clones and 51.8% over the matching clones. The remaining sequences are transcripts which have no match with databases sequences (24. 6% over total clones, with 542 clusters and 741 clones).

Table 1 shows the twenty most abundant groups of transcripts, with eight, related to 'known toxins' and 'possible toxins' products, eleven with 'cellular proteins', and one group, which is the most expressed, has non-match sequences (24.6% over total clones).

**Table 1:** Identification of the high abundance transcripts present in *L. laeta *venom gland

Groups	Number of clusters	Number of clones	Clones/clusters	% of total	Putative identification
1	542	741	1.37	24.6	No Hit*
2	91	489	5.37	16.3	Sphingomyelinase D*
3	59	248	4.20	8.2	Metalloproteinase*
4	83	105	1.27	3.5	Unknown Function*
5	9	75	8.33	2.5	Troponin
6	4	55	13.75	1.8	Actin
7	14	46	3.29	1.5	Hsp*
8	7	43	6.14	1.4	Elongation Factor
9	7	41	5.86	1.4	Salivary protein*
10	3	38	12.67	1.3	5'-nucleotidase*
11	7	36	5.14	1.2	Myosin
12	5	26	5.20	0.9	Chitinase*
13	4	23	5.75	0.8	Tubulin
14	2	23	11.50	0.8	LIM protein
15	6	22	3.67	0.7	PDI*
16	2	18	9.00	0.6	Venom allergen III*
17	6	15	2.50	0.5	Lectin*
18	12	14	1.17	0.5	Serine Protease*
19	2	13	6.50	0.4	ATP-binding
20	9	12	1.33	0.4	Ubiquitin

The (*) designates the detection of a putative signal peptide, predicted by using SignalP 3.0 program [54].

### ESTs relevant to cellular functions (Cellular Proteins)

**Figure 2:**
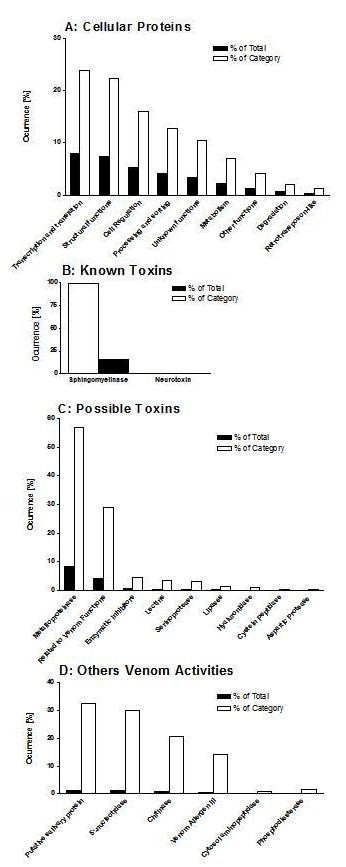
Representation of the groups 'Cellular Proteins' [A], 'Known Toxins' [B], 'Possible Toxins' [C] and 'Others Venom Activities' [D], a subgroup of 'Possible Toxins', from the transcriptome of *Loxosceles laeta *spider venom glands, showing the relative distribution of the transcripts, proportions over transcripts (■) and over category (□).

From a total of 751 clusters that presented significant hits in the databases, 33.3% represent proteins involved in various cellular functions. Figure 2A shows that 'gene transcription and translation proteins' are the most abundant transcripts in this category (8.0%), which may reflect the functional feature of this specialized tissue in the production and secretion of substances involved in feeding and predator interactions. The majority of the transcripts, involved in transcription and translation functions, are ribosomal proteins; the translation initiation and elongation factors (such as the group 8, from Table 1) and ATP-binding proteins (group 19) are also highly expressed.

'Proteins related to structural functions' account for 7.4% of the total of sequences, being the high expressed groups 5, 6, 8 and 11 (Table 1) among this category. Cellular regulation transcripts account for 5.3%, being the group 14 (Table 1) the most abundant of this category, with sequences highly similar to LIM protein (gb|AAV91410.1).

'Processing and sorting of proteins' account for 4.3% of the sequences and, the majority of the transcripts, are related to Heat shock protein (Hsp) and Protein disulfide isomerase (PDI), as can been seen in Figure 2A and Table 1 (groups 7 and 15). The most abundant cluster of this category, LLAE0312C (containing 26 clones), is similar to heat shock protein 20.6 from *Locusta migratoria *(gb|ABC84493.1), with 66% of identity. The main cluster matching PDI, LLAE0141C (containing 17 clones), is similar to ER protein disulfide isomerase from *Aedes aegypti *mosquito (56% identity). PDI proteins have also been described in the saliva of the ticks, as reported by KNIZETOVA *et al*. (2006) that isolated a new member of the PDI family in *Amblyomma variegatum*. The present study is the first reporting the presence of Hsp and PDI proteins in the venom glands of spiders. These transcripts are targets for further isolation, functional characterization and evaluation of their pharmacological properties.

'Other functions' account for 1.4% of total sequences, and includes ESTs related to complement components [LLAE0374S similar to complement component 3-like protein (*Carcinoscorpius rotundicauda*) (gb|AAQ08323.1), LLAE0774S similar to prominin-like protein (*Danio rerio*) (ref|XP_684527.1) and LLAE0889S similar to complement factor B (*Strongylocentrotus purpuratus*) (ref|NP_999700.1)], coagulation factors [LLAE0712S similar to echinonectin (*Lytechinus variegatus*) (gb|AAC32598)] and cell adhesion molecules [LLAE0711S similar to immunoglobulin domain cell adhesion molecule (*Rattus norvegicus*) (ref|XP_001070255.1)] (Figure 2A). These molecules may be involved in the haemolymph maintenance, as they are observed in other arthropods, and there are no reports about the presence of them in *Loxosceles *venom. Nevertheless, complement and clotting factors should always be regarded as possible disturbing elements.

'Degradation of peptides' account for 0.7% of the total number of sequences; most of them are represented by ubiquitin, group 20 (Table 1). An interesting aspect is the presence of sequences showing homology with retrotransposable elements, including transposases and reverse transcriptases. The remaining 83 clusters, corresponding to 3.5% of total clones, were classified as 'unknown function', being similar to conserved hypothetical or unknown proteins, in nr database, with unknown functions, as represented in group 4 (Table 1).

### ESTs relevant to the envenomation process (Known Toxins)

From a total of 751 clusters presenting significant hits in databases, 16.4% represent proteins related to toxic functions, including sphingomyelinases D (489 clones and 93 clusters) and neurotoxins (5 clones and 2 clusters) (Figure 2B).

The predominance of sphingomyelinase D clones is not surprising, since it has already been described as the central toxic component of *Loxosceles *spider venom [12,14,19-22]. We have recently obtained the first crystal of a SMase D from *Loxosceles laeta *and solved its structure [23,24]. All the spider venom SMases D sequenced to date display a significant level of sequence similarity and thus likely possess the same (α/β)_8 _or TIM barrel fold [24,25]. These new sequences described in this paper will help us to further investigate the diversity and structural/functional aspects of the SMases D present in *Loxosceles laeta *venom glands.

Figure 2B also shows the presence of neurotoxins, corresponding to 0.2% of the total matched sequences. The transcripts are similar to the neurotoxin magi-3 from *Macrothele gigas *(sp|P83559|TXMG3). This is a wild spider, living in forests, and no envenomation reports have been associated to this animal. Neurotoxins are important tools for predation and defence strategies and they, probably, are present in most spider venom glands. Interestingly, FOIL *et al*. (1979) have partially characterized lethal and neuroactive components in *Loxosceles reclusa *venom. The detection of these neurotoxins sequences in the cDNA library will allow us to evaluate their role in the genesis of loxoscelism.

### ESTs possibly related to toxic functions (Possible Toxins)

Sequences, for which it was possible to assume a toxic potential, were included in the category 'possible toxins'. This group is represented by 14.5% of the significant hits in the databases, with 435 clones and 117 clusters (Figure 2C).

The transcripts presenting similarity to 'metalloproteinases' account for 8.3% of the total sequences, as present in group 3 of Table 1. The cluster LLAE0224C corresponds to the most abundant cluster, with 25 clones matching to astacin protease family member (nas-37) from *Caenorhabditis elegans *nematode (ref|NP_001024413.1). Although some reports have already described the presence of metalloproteases in *Loxosceles *sp venoms [27,28], the present data are interesting not only because of the high number of transcripts similar to metalloproteinases found, but also for the possibility of developing studies in order to characterize these molecules and their role in the loxoscelism.

Transcripts similar to 'serinoproteinases', correspondent to 0.5% of the total sequences and 3.2% of this category, were identified (Figure 2C) and are represented in group 18 of Table 1. High molecular weight serinoproteinases of 85- and 95-kDa have been previously identified in *L. intermedia *venom and considered as toxic factors [29]. Sequences similar to cystein peptidases (2 clones/2 clusters), lipases (6 clones/5 clusters) and aspartic proteases (1 clones/1 cluster) were also identified, representing 0.3% of the total hits (Figure 2C). The cystein peptidases present in this library have homology with proteins that regulate the autophagic system [APG4 (ref|NP_998738.1)] and degradation of proteins [ubiquitin thiolesterase (gb|AAI10248.1)]. Therefore, they could perform either a physiological role or toxic (predation/digestion) functions in the venom glands, what is suggested by the presence of signal peptide in the cystein peptidase LLAE0692S of our library, while the proteins APG4 and ubiquitin thiolesterase do not present signal peptide. Though analysis of lipases and aspartic proteases ESTs were not found similar proteins endowed with toxic functions, we can not exclude that this molecules perform others roles beyond physiological functions.

Sequences matched with 'enzymatic inhibitors' were also detected and represent 0.6% of the total number of sequences (19 clones/11 clusters) (Fig. 2C). The singlets LLAE0371S and LLAE0438S are related to the intracellular coagulation inhibitor from *Tachypleus tridentatus *arthropod (dbj|BAA12795.1), while the LLAE0785S, LLAE0391S, LLAE0635S, LLAE0965S and LLAE1134S are similar to serine (or cysteine) proteinase inhibitors from *Mus musculus *(ref|NP_033152.2), *Aedes aegypti *(gb|EAT35458.1), *Branchiostoma lanceolatum *(emb|CAD68157.1), *Gallus gallus *(ref|XP_421343.1) and *Boophilus microplus *(gb|ABG36931.1). Cystatins are biochemically well-characterized as strong inhibitors of cysteine proteinases of the papain protease family, especially cathepsins, and also of some lysosomal caspases, such as legumain. An important contribution of cystatins in the regulation of the cysteine proteinases is probably the control of intracellular protein degradation [30]. The Bmcystatin, a fat body cysteine proteinase inhibitor from the tick *Boophilus microplus*, was cloned and characterized as C1 cysteine peptidase inhibitor, with Mr of 11 kDa and pI 5.7 [31]. The presence of these transcripts in the *L. laeta *cDNA library will allow the isolation and characterization of these putative inhibitors.

A group of 'C-type lectin' (0.5% of the total) was also detected, group 17 with 15 clones and 6 clusters (Table 1), which represents 3.5% of this category (Figure 2C). Group 17 in Table 1 (LLAE0029C, LLAE0069C, LLAE0091C, LLAE0139C, LLAE0589S and LLAE0596S) matches to C-type lectins from the *Bos taurus *(mammal), *Tachypleus tridentatus *(arthropod), *Bombyx mori *(insect) and *Sus scrofa *(mammal), respectively. C-type lectins are proteins of animal origin, calcium-dependent, that bind carbohydrates. Animal C-type lectins are involved in extracellular matrix organization, endocytosis, complement activation and also mediate pathogen recognition and cell-cell interactions [32]. A lectin-like peptide was isolated from the venom of the Chinese bird spider *Selenocosmia huwena*, and the biological activity assays showed that this peptide has very low toxicity to both mammals and insects, though is abundantly present in the venom [33]. Lectins are also present in venoms of various snakes and other poisonous animals [34].

Aiming at to stand out the importance of these molecules, as future interest to isolate new peptides or proteins in order to characterize their role in the loxoscelism, as well as their pharmacological or biotechnological applications, we aligned amino acid sequence of some selected clusters from hyaluronidases, 5'-nucleotidases, chitinases and venom allergens with known sequences in database. Some discussion about sequence features of these molecules is provided below.

**Figure 3:**
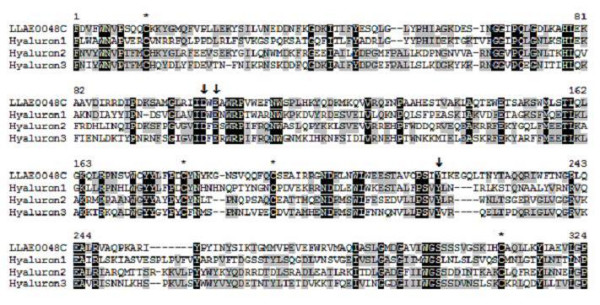
**Alignment of the amino acid sequence of LLAE0048C, from *L. laeta *venom glands, with known hyaluronidases.** Residues are numbered according to the aligned hyaluronidases sequences and dots represent gaps introduced to improve alignment. The conserved cystein residues are indicated by asterisks. Black and gray indicate amino acids that are identical or conserved, respectively. The three key catalytic residues are represented by arrows. The abbreviation and *GenBank *accession number for the hyaluronidases sequences aligned are: hyaluron1, *Bos taurus *hyaluronidase (AAP55713); hyaluron2, *Apis mellifera *hyaluronidase (AAA27730) and hyaluron3, *Vespula germanica *hyaluronidase (CAL59818).

Transcripts with similarity to 'hyaluronidase' from *Bos taurus *(gb|AAP55713.1), were also found, with 4 clones and 1 cluster (LLAE0048C), representing 0.13% of the total sequences. Hyaluronidases are ubiquitously expressed enzymes that naturally cleave hyaluronic acid, which is a major component of the extracellular matrix of vertebrates [35]. *Loxosceles *venom hyaluronidases were previously described as molecules with, 33 and 63 kDa in *L. reclusa *[36], 32.5 kDa in *L. rufescens *[37] and 44 kDa in *L. deserta*, *L. gaucho*, *L. intermedia*, *L. laeta *and *L. reclusa *[38]. Hyaluronidases in *Loxosceles *venom have been characterized as spreading factors, increasing the diffusion of other toxins and also contributing to the gravitational dissemination of the local reaction [1]. Figure 3 shows the alignment of the deduced amino acid sequence of LLAE0048C from *L. laeta *venom glands with known hyaluronidases from *Bos taurus*, *Apis mellifera *and *Vespula germanica*. The highest similarity between *L. laeta *hyaluronidase sequences and those present in *GenBank*, is in the conserved cystein domain and the three key catalytic residues [39], suggesting that LLAE0048C from *L. laeta *encodes hyaluronidase enzymes.

We have also established a subgroup of clusters matching proteins (possible toxins) that has never been observed, isolated or characterized in spider venom glands, denominated 'Others venom activities', to describe transcripts resembling putative salivary protein, 5'-nucleotidases, chitinases and venom allergens, that represent 4.2% of the total sequences, with 126 clones and 20 clusters (Table 1, Figure 2D).

The main group of transcripts, with similarity to a 'salivary protein' (gb|AAY66605.1), is composed by 41 clones and 7 clusters (group 9/Table 1 and Figure 2D). The majority of these transcripts was similar to proteins from *Ixodes scapularis *tick, a arachnid that may use these proteins of saliva for feeding functions, since saliva of blood-sucking animals contains powerful substances able to prevent blood clotting during their feeding [40].

**Figure 4:**
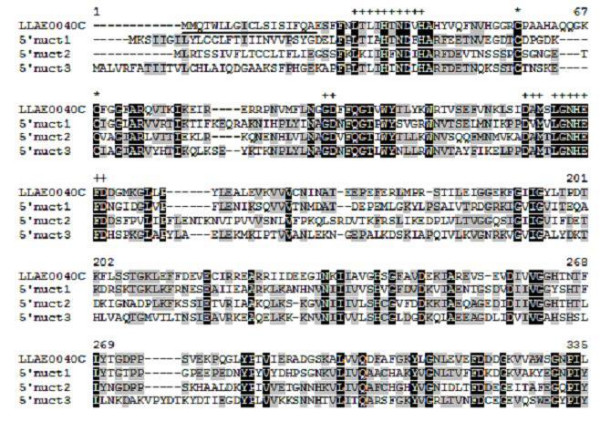
**Alignment of the amino acid sequence of LLAE0040C, from *L. laeta *venom glands, with known 5'-nucleotidases.** Residues are numbered according to the aligned 5'-nucleotidase sequences and dots represent gaps introduced to improve alignment. The underlined amino acid sequence indicates the putative signal peptide. The conserved cystein residues are indicated by asterisks. Black and gray indicate amino acids that are identical or conserved, respectively. The 5'-nucleotidases active site regions are indicate by crosses. The abbreviation and *GenBank *accession number for the 5'-nucleotidases sequences aligned are: 5'nuct1, *Glossina morsitans morsitans *5'-nucleotidase (ABN80093); 5'nuct2, *Aedes aegypti *5'-nucleotidase (ABF18486) and 5'nuct3, *Anopheles gambiae *5'-nucleotidase (CAB40347).

A group of transcripts related to '5'-nucleotidase' (1.3% of the total) was also detected with 38 clones and 3 clusters (group 10, Table 1; Figure 2D). The LLAE0040C (36 clones), LLAE1315S and LLAE1233S are the main sequences, matching with proteins from *Boophilus microplus *(gb|AAB38963.1), *Strongylocentrotus purpuratus *(ref|XP_794802.1) and *Xenopus laevis *(gb|AAH97618.1), respectively. The ecto-5'-nucleotidases are a widely distributed group of enzymes, hydrolysing a variety of nucleoside mono-, di- and triphosphates to release the free nucleoside. This enzyme is known to affect haemostasis by inhibiting platelet aggregation, since it depletes the ADP from plasma. The 5'-nucleotidases have been studied in detail in many organisms, particularly mammalian cells and tissues, but the amount of information available for insects and other arthropods is relatively small [41]. Figure 4 shows the alignment of the amino acid sequence of LLAE0040C from *L. laeta *venom glands with known 5'-nucleotidases from *Glossina morsitans morsitans*, *Aedes aegypti *and *Anopheles gambiae*. The high sequence similarity to known 5'-nucleotidases, the presence of putative conserved cystein residues and 5'-nucleotidase domains [42], indicate that LLAE0040C may encodes a 5'-nucleotidase enzyme.

**Figure 5:**
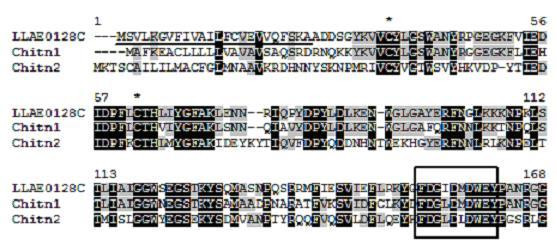
**Alignment of the amino acid sequence of LLAE0128C, from *L. laeta *venom glands, with known chitinases.** Residues are numbered according to the aligned chitinase sequences and dots represent gaps introduced to improve alignment. The underlined amino acid sequence indicates the putative signal peptide. The conserved cystein residues are indicated by asterisks. Black and gray indicate amino acids that are identical or conserved, respectively. Chitinase glycosyl hydrolases family 18 active site signature is marked by a box. The abbreviation and *GenBank *accession number for the chitinase sequences aligned are: Chitn1, *Araneus ventricosus *chitinase (AY120879) and Chitn2, *Dermatophagoides pteronyssinus *chitinase (DQ078740).

**Figure 6:**
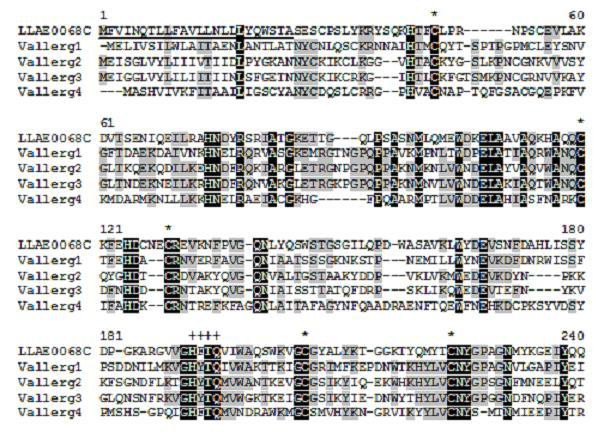
**Alignment of the amino acid sequence of LLAE0068C, from *L. laeta *venom glands, with known sequences of venom allergen III.** Residues are numbered according to the aligned of venom allergen III sequences and dots represent gaps introduced to improve alignment. The underlined indicates the putative signal peptide. The conserved cystein residues are indicated by asterisks. Black and gray indicate amino acids that are identical or conserved, respectively. The conserved motif HYTQ (residues 192–195) is indicated by crosses. The abbreviation and *GenBank *accession number for the venom allergen III sequences aligned are: Vallerg1, *Solenopsis invicta *(P35778), Vallerg2, *Vespula vulgaris *(Q05110), Vallerg3 *Dolichovespula maculata *(P10736.1) and Vallerg4 *Aedes aegypti *(EAT48176).

We have also detected transcripts matching with 'chitinases', representing 0.9% of total hits (Figure 5), with 26 clones and 5 clusters (Table 1). These ESTs show similarity to database proteins from *Araneus ventricosus *spider (LLAE0128C, LLAE0033C, LLAE0027C) (gb|AAN39100.1), *Tribolium castaneum *(LLAE1239S) (gb|ABG47446.1) and *Bombyx mori *(LLAE0611S) (gb|ABF51237.1) insects. Chitin is one of the most abundant polysaccharides in nature and it is a linear polymer of β (1→4) linked to N-acetylglucosamine (GlcNAc) residues. It is one of the most unique biochemical constituents found in the exoskeletons and gut linings of arthropods and fungi. Chitinolytic enzymes that catalyse the hydrolysis of chitin have been found in chitin-containing organisms, as well as in microorganisms that do not have chitin. The chitinases from various organisms have many biological functions: they play roles in the moulting process of invertebrates, including spiders, the digestion of chitinous food, and defence against chitin-bearing pathogens [43]. HAN *et al*. (2005) cloned a fat body-specific chitinase cDNA from the spider *Araneus ventricosus*; the cDNA was expressed as an active chitinase enzyme with 61 kDa. Figure 6 shows the alignment of the amino acid sequence of the main cluster similar to chitinase from *L. laeta *venom glands (LLAE0128C), with known chitinases from *Araneus ventricosus *and *Dermatophagoides pteronyssinus*. The high level of similarity between the sequences from *L. laeta *to chitinases and the presence of the active site [44] suggests that LLAE0128C encodes a transcript with chitinase enzymatic activity.

The transcripts similar to 'venom allergen III' (sp|P35779|VA3_SOLRI) represent 0.6% of the total sequences (Figure 2D), with 18 clones and 2 clusters, as showed in group 16 (Table 1). The LLAE0068C (17 clones) and LLAE1344S matched with databases proteins from *Solenopsis richteri *and *Solenopsis invicta *insects, respectively. Venoms of *Hymenoptera*, including vespids, honey bees and fire ants are common causes of allergic reactions. Venom from the red fire ant, *Solenopsis invicta*, contains four important potent allergens, which cause anaphylactic reactions in highly sensitive individuals. Figure 6 shows the alignment of the amino acid sequence of LLAE0068C from *L. laeta *venom glands with known sequences of venom allergen III from *Solenopsis invicta*, *Vespula vulgaris*, *Dolichovespula maculata *and *Aedes aegypti*. The similarity of the putative amino acid sequence of LLAE0068C from *L. laeta *to known venom allergen III sequences, including the presence of conserved cystein residues and the motif HYTQ (residues 192–195), which is shared by the majority of the allergen-like proteins [45], suggests that spider venom may possess venom allergen III-like proteins.

### New transcripts that may encode venom toxins (No Hit)

'No hits' represents the biggest group of transcripts analysed in *L. laeta *cDNA library, accounting for 24.6% of total sequences, with 741 clones and 542 clusters (Figure 1 and Table 1). This result is not surprising, since there is a limited number of annotated *Loxosceles *spider nucleotide sequences currently deposited in the public databases. 320 transcripts (59%) of the 542 'non- hits' sequences contain putative signal peptide, as determined by using SignalP 3.0 program (data not shown). The search for conserved domains in databases (CDD) showed that most of the sequences have no function domains, except for a few that contain domains related to metabolism (data not shown).

The abundance of these transcripts, the failure of matching to known sequences and the presence of signal peptide suggest that may encode for novel toxins.

## Conclusion

By using Expressed Sequencing Tag strategy it was possible to reveal, for the first time, the transcript repertoire of a spider venomous gland. Results presented here show an ample range of structural and functional putative molecules in the gland of *Loxosceles *spider. *L. laeta *female spider specimens were milked to stimulate the production of mRNAs in the venom glands and after 5 days, the spider venom glands were used. This process was developed strategiclly to induce the majority possible toxins production. Sphingomyelinases D, the central toxin responsible for the main local and systemic reactions induced by the venom, corresponds to 16.4% of the sequences present in *L. laeta *gland, confirming the high representation of these proteins in the total transcript. Other transcripts presenting similarities to the sequences deposited in GenBank, may act as toxins, such as neurotoxins, hyaluronidases, metalloproteinases, lipases, serinoproteinases, C-type lectin, enzymatic inhibitors, cystein peptidases and others. This report also revealed the existence of transcripts related to others venom activities, including salivary proteins, 5'-nucleotidases, chitinases and venom allergens. Moreover, we found a high percentage of transcripts (25% of total sequences) that do not have any significant database matches, which opens up new avenues for exploration. Finally, these transcripts will be important tools not only for further investigate the molecular mechanisms of these spider proteins, as well as to uncover molecules with biotechnological potential.

## Methods

### Loxosceles laeta spider

*Loxosceles laeta *spiders were provided by "Laboratório de Imunoquímica, Instituto Butantan, SP, Brazil".

### Library construction

One hundred *L. laeta *female spider specimens were milked to stimulate the production of mRNAs in the venom glands. After 5 days, the spider venom glands were collected and kept in liquid nitrogen until use. For total RNA extraction, Trizol reagent (Gibco-BRL Life Technologies) was used according to manufacturer's protocol. An oligo (dT) cellulose column (Amersham) was used for mRNA purification. The cDNAs were synthesised from 5 μg mRNA using the Superscript Plasmid System for cDNA Synthesis and Cloning (Gibco-BRL Life Technologies), linked to *Eco*RI adapters (Amersham), and cloned in pGEM11Zf^+ ^plasmid (Promega) at *Eco*RI/*Not*I sites. *Escherichia coli *DH5α cells were transformed with the cDNA library plasmids and then plated on 2YT (Gibco-BRL Life Technologies) agarose plates containing 100 mg/mL ampicillin [14,46].

### EST sequencing, data processing and bioinformatics analysis

For large-scale DNA sequencing (EST generation), random clones were grown in antibiotic selective medium for 22 h and plasmid DNA was isolated using alkaline lysis [46]. The DNA was sequenced on an ABI 3100 sequencer, using BigDye2 kit (Applied Biosystems, Foster City, CA) and the standard M13 forward primer, rising 5' ESTs. Base-Calling was performed with PHRED and the cutoff Phred score was 20 [47]. Original sequences were processed by removing vector, adaptors and *E. coli *DNA sequences using CrossMatch [48]. High-quality ESTs were assembled into contigs, using the CAP3 program [49] set to join only sequences with at least 98% of base identity. To assign annotation to the assembled ESTs (clusters), these sequences were searched against the nr and nt (E values < 1e-05) for homologous comparison using BLASTX and BLASTN [50]. The metadata available for the first five hits, as well as bibliographic information when available, were manually inspected to assign the putative functional classification of the cluster. Categories used were based on those from Adams et al., 1995, [51] modified to fit in a toxin producing model. Additionally, the proteins coded by the clusters were grouped according to a possible participation in the venom. Three categories were created, 'known toxins', 'possible toxins' or 'cellular proteins', respectively for proteins with best hits to well-known toxins from spider venom, proteins with hits to non-spider toxin sequences presenting activities compatible with toxic actions of venoms, and other products related to cellular functions, without evidence of being toxins. The presence of conserved domains, using the nr protein database or the SMART [52] and Pfam [53] was also used to guide the functional attribution. The occurrence of signal peptide was predicted with the SignalP 3.0 program [54], using both Neural Networks (NN) and Hidden Markov Models (HMM) methods. A secretory protein was considered when both methods showed it possessing a signal peptide according to their default parameters (mean S > 0.048 and mean D score 0.43 > in NN and signal peptide probability > 0.5 in HMM).

## Authors' contributions

MdFF–P performed the cDNA library, bioinformatics analysis and drafted the manuscript. IdLMJ–d–A performed the data processing, bioinformatics analysis and drafted the manuscript. RMG–d–A identified, collected the spiders, isolated the venom glands and drafted portions of the manuscript. LSK performed the minipreps and DNA sequencing. DDA performed partial bioinformatics analysis and drafted portions of the manuscript. PLH participated in data analyses resulting and drafted portions of the manuscript. DVT conceived and coordinated the study and contributed in project design, interpretation and drafted the manuscript. All authors read and approved the final manuscript.
